# Spatial Distribution of the Risk of Dengue and the Entomological Indicators in Sumaré, State of São Paulo, Brazil

**DOI:** 10.1371/journal.pntd.0002873

**Published:** 2014-05-15

**Authors:** Gerson Laurindo Barbosa, Maria Rita Donalísio, Celso Stephan, Roberto Wagner Lourenço, Valmir Roberto Andrade, Marylene de Brito Arduino, Virgilia Luna Castor de Lima

**Affiliations:** 1 Superintendence for Control of Endemic Diseases, State Health Department, São Paulo, São Paulo, Brazil; 2 Department of Public Health, Faculty of Medical Sciences, State University of Campinas, Campinas, São Paulo, Brazil; 3 Department of Environmental Engineering, State University of São Paulo, Sorocaba, São Paulo, Brazil; Centers for Disease Control and Prevention, Puerto Rico, United States of America

## Abstract

Dengue fever is a major public health problem worldwide, caused by any of four virus (DENV-1, DENV-2, DENV-3 and DENV-4; *Flaviviridae*: *Flavivirus*), transmitted by *Aedes aegypti* mosquito. Reducing the levels of infestation by *A. aegypti* is one of the few current strategies to control dengue fever. Entomological indicators are used by dengue national control program to measure the infestation of *A. aegypti*, but little is known about predictive power of these indicators to measure dengue risk. In this spatial case-control study, we analyzed the spatial distribution of the risk of dengue and the influence of entomological indicators of *A. aegypti* in its egg, larva-pupa and adult stages occurring in a mid-size city in the state of São Paulo. The dengue cases were those confirmed by the city's epidemiological surveillance system and the controls were obtained through random selection of points within the perimeter of the inhabited area. The values of the entomological indicators were extrapolated for the entire study area through the geostatistical ordinary kriging technique. For each case and control, the respective indicator values were obtained, according with its geographical coordinates and analyzed by using a generalized additive model. Dengue incidence demonstrated a seasonal behavior, as well as the entomological indicators of all mosquito's evolutionary stages. The infestation did not present a significant variation in intensity and was not a limiting or determining factor of the occurrence of cases in the municipality. The risk maps of the disease from crude and adjusted generalized additive models did not present differences, suggesting that areas with the highest values of entomological indicators were not associated with the incidence of dengue. The inclusion of other variables in the generalized additive models may reveal the modulatory effect for the risk of the disease, which is not found in this study.

## Author Summary

Dengue is a disease caused by a virus which has four serotypes DENV-1, DENV-2, DENV-3 and DENV-4. In Americas, *A. aegypti* is acknowledged as the only dengue vector in America. Currently the only strategy to prevent dengue is controlling *A. aegypti* mosquitoes. The generalized additive model was used to understand the relationship of the indicators of the presence of eggs, larvae-pupae and adult stages of *A. aegypti* with the occurrence of dengue cases in a medium sized city of São Paulo state, Brazil. Dengue incidence as well as the entomological indicators in all stages of the mosquito showed a seasonal behavior. The infestation level was not a limiting or a determinant factor of the occurrence of cases in the municipality. Risk maps of the disease, from the crude and adjusted by generalized additive models, showed no differences, suggesting that the entomological indicators did not influence the incidence of dengue in the city. The inclusion of other variables in the generalized additive model could reveal the modulating effect on the disease risk, not found in this study.

## Introduction

Dengue is a disease caused by any of four virus (DENV-1, DENV-2, DENV-3 and DENV-4; *Flaviviridae*: *Flavivirus*) and has become a major global health issue. An epidemic similar to dengue was registered in 1699 in Central America. In Philadelphia, USA, a major epidemic occurred in 1780 and epidemics became common in the early 20th century [1].

There have been references of dengue epidemics, in Brazil, since 1923 but with no laboratorial confirmation until 1986. Dengue fever outbreaks have occurred in several states since 1986, with the identification of the DENV-1 virus in 1986 [2], DENV-2 in 1990 [3], DENV-3 in 2001 [4] and DENV-4, isolated in Manaus from 2005 to 2007 [5] and in São Paulo in 2011 [6]. All four serotypes are currently circulating in Brazil [6]. In 2010, with over one million cases of the disease, the highest reported incidence occurred in the Northern and Central-Western regions, with 621.7 and 1,536.8 cases per 100,000 inhabitants, respectively. In these regions, the majority of municipalities presented rates higher than 300 per 100,000 inhabitants [7], [8]. In the state of São Paulo, the incidence in 2010 was of 503 cases per 100,000 inhabitants [8].

*A. aegypti* is still the only vector of epidemiological importance as the transmitter of the dengue virus (DENV) in the Americas [9], [10]. Reducing levels of *A. aegypti* infestation is one of just a few strategies for disease control, since there is no vaccine available yet. Other undirected strategies refer to basic sanitation, garbage collection and proper water supply, which would eliminate the need for water storage. Besides, we have education activities to improve population commitment in order to eliminate breeding sites.

The dengue national control program uses sampling methods to collect data in the field and to build indicators of *A. aegypti* presence, in the various stages of the vector life cycle, mainly traditional Breteau index and House index [11]. Although adult forms have a direct impact on virus transmission, the most used indicators to measure vector infestation are based on larvae, pupae and eggs [12].

Some studies demonstrate the direct relationship among infestation levels with the risk of epidemics in various regions of the world [13]–[16], though epidemic transmission is also reported in the presence of very low infestation levels [17]–[20].

In addition, factors associated to human population organization have a decisive role in the circulation of the virus and in the establishment of breeding sites of *A. aegypti* [21], which has demonstrated a great ability to adapt to different environmental contexts [9], [22], [23]. The multiple factors involved in the transmission of the disease therefore, require different approaches for understanding the forms of transmission.

Over the last few decades, geoprocessing and digital mapping techniques were incorporated to the analysis of public health issues, as well as the use of spatial analysis programs to visualize the spatial distribution and spatio-temporal patterns of epidemiological data [24], [25]. These techniques allow the development of models to predict the risk of disease and territorial infestation, mapping environmental and social conditions associated to such patterns [26]. A number of studies using these techniques have analyzed the spatial and temporal distribution of *A. aegypti* [27], [28] and dengue transmission [29]–[32], as well as their relationships between each other [33], [34].

One of the main instruments for the operationalization of the vector control program is by monitoring the dispersion and abundance of mosquitoes through entomological indicators. It would be useful to understand the spatial and temporal patterns in small geographical scales [35]. These indicators are currently part of service routines in Brazil, but suffer from a number of limitations and particularities identified from control program perspective, which have not been studied yet.

This study has the objective of evaluating the association between the spatial distribution of incidence of dengue and the entomological indicators in a middle-size city in the state of São Paulo, Brazil.

## Methods

**Figure 1:**
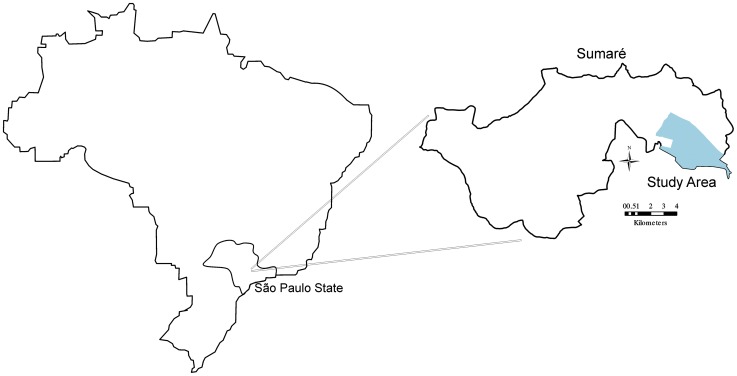
Municipality of Sumaré, São Paulo state, Brazil. Legend of Figure 1: Municipality located in state of São Paulo, southeast region of Brazil. The area studied is shown highlighted.

This spatial case-control study analyzed the risk of dengue virus transmission and its association with entomological indicators of *A. aegypti* in Sumaré, São Paulo. The study area is a domiciliary district with 63 census tracts and 51,253 inhabitants (Brazilian Institute of Geography and Statistics, 2010), and presents the highest incidence of the disease in the municipality (Figure 1). The prevalence of dengue cases in the area has been reported since 1997 and vector infestation since 1994. Sumaré, located at latitude 22°49′19″S and longitude 47°16′01″W, is the second largest city in the Campinas metropolitan area, in the state of São Paulo, with a population of approximately 240,000. It is situated at an altitude of 583 m and has a highland tropical climate (Köeppen Geiger classification) [36], with a temperature range of 17.8°C to 25.5°C and a cumulative annual rainfall of 1371.8 mm; the period of October to March is the warmest and most humid period.

The municipality has been facing the problem of dengue transmission over the last 12 years, with the highest incidence rates of 1382, 532 and 506 cases per 100,000 inhabitants recorded in 2007, 2010, and 2011, respectively. This prevalence of dengue transmission highlights the deficiencies in urban public sanitation services an HDI (Human Development Index) of 0.762 (2010) besides the intense traffic of workers and students in the study area. DENV-1, DENV-2, and DENV-3 have already been isolated in Sumaré.

We included all laboratory confirmed and reported dengue cases according to clinical epidemiological criterion [37], considering the date of the onset of the symptoms, that were recorded in the Information System on Diseases of Compulsory Declaration (SINAN), from January to September of 2011 (n = 195). The dengue cases were georeferenced using a portable global positioning system (GPS) device, thus obtaining the geographic position of all dengue cases.

To evaluate the association of the spatial distribution of entomological indicators with the spatial distribution of dengue cases in the area, we generated randomly 1000 points inside the inhabited area (63 census tracts) that correspond to controls. We assumed that these points correspond to spatial location of individuals without the disease, choosing 1 control for 51 inhabitants. The geographical distribution of controls was weighted according to the population within each census tract. The control group represented the spatial distribution of the source population of cases; the entire process was conducted using the Arcmap 10.0 program.

The vector indicators were built by collecting the four life stages of *Aedes aegypti* in the study area.

The three indicators used were as follows:

Egg Indicator: number of *A. aegypti* eggs in the block (absolute number)
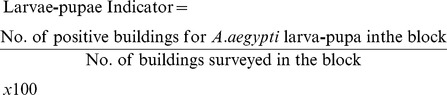





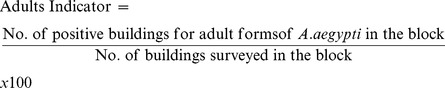



The study vector population was composed by all buildings in the area. The sample was built by cluster probabilistic, where the block was the first unit of draw. The catch of adult forms was done in two stages, where the block was the first stage and buildings the second [38], [39]. The catch of larvae-pupae draw was done in one stage, where all buildings in the block were visited. The catch of eggs was done in one stage but the buildings where the trap was placed were selected in a non-probabilistic way.

The first block of the sample was randomly selected and the others were systematically selected by adding the sample interval in order to complete the sample number [39].

In each activity we worked monthly in 14 blocks. A total of 400 buildings were visited in the larvae-pupae catch and one in every three (1/3) buildings were visited to perform the adult forms catch. To collect the eggs four traps were placed in two buildings in every block one inside and the other outside the house. All the traps were surveyed every five days in accordance with the minimum development time of the biological cycle of the mosquito and thus avoiding them to configure breeding sites [38], [39].

In the catch of larvae-pupae the presence of larvae or pupae of *Aedes aegypti* was investigated in all containers with water in households [40]. The collection of adult forms was performed indoors and outdoors through dip nets and Nasci aspirators [41]. We used oviposition traps for egg collection consisting of a black plastic material filled with tap water and hay infusion, with fiber chipboard for oviposition [42]. The collected eggs were placed for hatching for species identification.

In order to reduce the interference in the infestation area with periodic collection of various stages of the mosquito in the same block away and to avoid distortion in the evaluation of the infestation area, for each month and activity, it was made independent draw with replacement, i.e., all blocks were included in every draw.

All worked properties were georeferenced using GPS and the indicators were calculated for each block and the values have been assigned to their centroids. All points were identified on the digital map base.

For analysis we aggregated entomological indicators results in quarters for analyses consistency; the first quarter was February-March-April (Q1) and the following quarters were built by extracting the first month (February) and inserting the following (May) forming the quarter March-April-May (Q2) and so on to Q3 and Q4. The quarterly approach was used to give more consistency to entomological indicators results because we had only 14 collection data points each month.

The quarterly entomological indicator values of egg, larvae-pupae and adult forms for each point in the study area were estimated by a geostatistical interpolation technique called *ordinary kriging*, which uses local averages or local trends from sampled values. Ordinary kriging produces an spatial distribution estimate of a variable by taking into account adjacent values [43] and it uses the spatial dependence amongst neighboring samples, expressed in the semivariogram function [43], [44].

For each case and control spatial position, there was a value of each entomological indicator. Surface from where we obtained points values were generated by the kriging process for each quarter, using the *Extract Values to Table* tool, available in the *Arctoolbox* (ArcMap 10.0) software.

We adjusted a generalized additive model (GAM), taking into account the response variables, the case status (1), control (0), and the predictive variables, i.e., the entomological indicators, and the spatial component (geographical coordinates) according to the equation [45]:


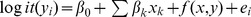
, where,


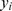
 is the response variable, i.e., the case or control status


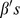
are the model coefficients,


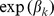
is the odds ratio


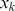
is the variable value,


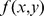
is the geographical coordinate's smoothing function and contains the “band width” parameter, in this case, the mosquito's flight radius, estimated to be 200 m [46] and


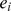
is the model's residue.

The advantage in use of GAM is that it describes the relationship between outcome and predictors without imposing specific parametric forms on the relationship. This method provides an unified framework for mapping case-control data, allowing spatial smoothing of binary outcome.

In order to adjust the model, the software R version 2.12.1 was used, with the epigam and vgam libraries. The epigam library was developed in the EpiGeo - Spatial Analysis Laboratory for Epidemiological Data of the Department of the Public Health from FCM/UNICAMP.

### Ethics Statement

The fieldwork was carried out with the consent of the residents, and no references were made to the names or addresses of the residents, dengue patients, and control individuals. This project was approved by the Research Ethics Committee - CEP/UNITAU, declaration No. 302/12, protocol 459/09.

## Results

From February to December of 2011, 4,688 buildings were inspected and *A. aegypti* larvae and/or pupae were found in 186 (4%) buildings. We surveyed 1,711 households for the presence of *A. aegypti* adult forms. We captured 582 specimens of *A. aegypti* in 17.7% of the domiciles. The percentage of adult forms was greater than that of immature forms in the buildings. We collected 11,395 eggs; 65.4% were in the peridomicile area and 34.6% in the intra-domicile.

**Figure 2:**
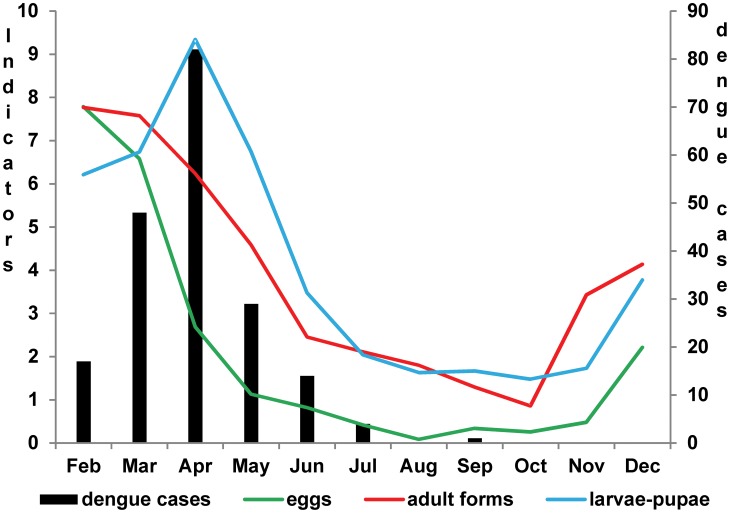
Dengue cases and entomological indicators in stages: egg, larvae-pupae and adult forms. Legend of Figure 2: The bar chart shows the number of dengue cases in the study area and the lines show the entomological indicators measured from February to December 2011. In order to compare the indicators seasonality in the same scale, the numbers of eggs were divided by 500 and the indicator of adult forms was divided by 5.

Whilst a larger number of eggs were found outdoors than indoors during the entire period, the number of eggs was larger indoors than outdoors during the driest months (Table 1). A similar seasonal pattern was observed for the three indicators during the hottest and wettest period of the year (February to April) (Figure 2). During this period, 195 dengue cases were reported, all of which practically occurred during the first semester, the hottest and most humid period of the year.

**Table 1:** Entomological indicators of *Aedes aegypti* stages: egg, larvae-pupae and adult forms, in the municipality of Sumaré-SP, from February to December 2011.

Month	Dengue Cases	Larvae-pupae Indicator	Adults Indicator	Eggs Indicator	% Eggs in peridomicile		% Eggs in intradomicile
Feb	17	6.2	38.8	3893		62.8	37.2
Mar	48	6.7	37.9	3290		77.7	22.3
Apr	82	9.3	31.2	1345		75.4	24.6
May	29	6.7	22.9	563		29.6	70.4
Jun	14	3.5	12.3	410		41.7	58.3
Jul	4	2.0	10.6	208		28.1	71.9
Aug	0	1.6	9.0	42		0.0	100.0
Sep	1	1.7	6.5	168		48.7	51.3
Oct	0	1.5	4.3	129		96.5	3.5
Nov	0	1.7	17.1	240		45.7	54.3
Dec	0	3.8	20.7	1107		65.6	34.4
Overall	195	4.0	17.7	11395		65.4	34.6

The dengue epidemic curve follows the larva-pupa indicator curve, where there is an increase up to April and a decrease up to the last case reported in September. We also observed a similarity between the curves for the adult forms and egg indicators, i.e., a decreasing curve up to the end of the dengue epidemic.

**Figure 3:**
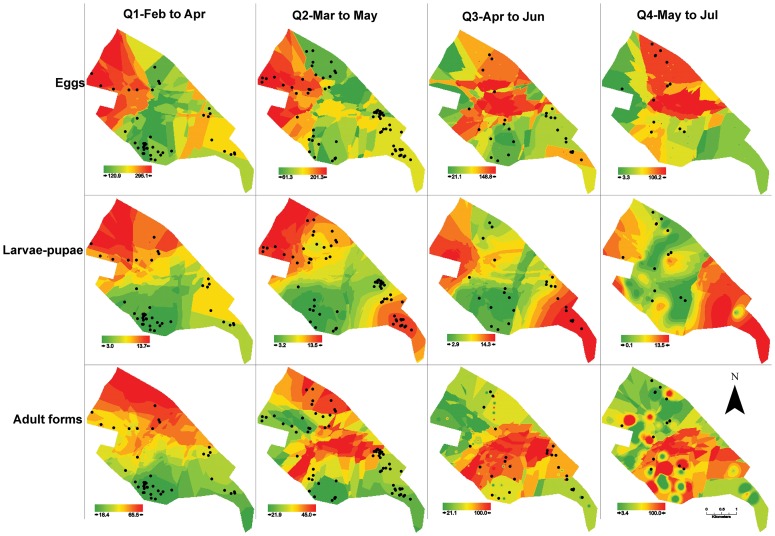
Ordinary Kriging maps of entomological indicators of *Aedes aegypti* and dengue cases. Legend of Figure 3: The figure shows the estimated ordinary kriging of entomological indicators from February to July 2011 divided in four groups quarterly. The cases that occurred in the month corresponding to the middle of the period by each cluster are also plotted as black dots in the maps. The color gradient, corresponding to the variation range of the estimated entomological indicators, is shown for each map. For eggs indicator the values represent to the number of eggs. For indicators of larvae-pupae and adult mosquitoes, the values match to the percentage of positivity of blocks.

Figure 3 presents the monthly dengue cases (points) and the quarterly entomological indicators estimated by the *ordinary kriging*, showing areas of different infestation intensity, with a gradient of colors varying from green (lower values) to red (higher values). We noticed a similarity among indices for all quarters, although some differences among the three indicators in the quarters can be pointed out. The Q2 quarter adult index did not agree with the larva-pupa and egg indices; in Q3 quarter, the larva and pupae indices did not agree with the adult and egg indices; in the last quarter, Q4, all the three indices were discordant (Figure 3).

We observed that during Q1, the cases in March were highly concentrated in the southern region of the map, where the three indicators presented low values. During Q2, the cases in April were highly concentrated in the eastern region, with low values for eggs and adult indicators. The intensity of the epidemic was reduced during Q3 and Q4, when dengue cases were reported all over the city, albeit with different patterns of infestation.

We observed the incidence of dengue as well as the presence of the vector in different stages of its biological cycle throughout the area during the entire period of the study. However, there was little spatial coincidence amongst the incidence of dengue and the intensity of the entomological indicators. The exceptions were the presence of case clusters and high adult infestation in Q2 and Q3, and also a degree of agreement between high levels of larva-pupa infestation and incidence of dengue in Q3 (Figure 3).

Table 2 shows the estimated risk of dengue and its respective confidence interval obtained from the adjusted model (GAM). We noted that in most of the analyzed period the highest values of the entomological indicators did not match with the dengue cases areas. An exception was viewed from February to April with the presence of larvae-pupae related with an elevated risk of disease. In the period from May to July the indicators related to eggs and larvae-pupae were associated with a mild risk of dengue. Egg indicator did not present statistical association with cases in the multiple model from February to April, so like the adult form indicator from April to June. All the entomological indicators presented negative association with the occurrence of dengue cases from March to May, showing that areas with elevated indicators presented minor occurrence of dengue cases in the period. Model results corroborating with those observed in Figure 3 in which higher occurrence of dengue cases did not match with higher entomological indicators.

**Table 2:** Dengue risk estimated obtained from the generalized additive model and confidence interval adjusted for the quarterly entomological indicators studied, Sumaré-SP, from February to July 2011.

stadium	Q1		Q2		Q3		Q4	
	OR*	CI**	OR*	CI**	OR*	CI**	OR*	CI**
eggs	0,99	0,98–1,01	0,96	0,95–0,96	1,01	0,98–1,04	1,06	1,03–1,09
larvae-pupae	3,41	2,59–4,48	0,51	0,48–0,55	0,75	0,68–0,82	1,43	1,22–1,68
adult forms	0,91	0,86–0,95	0,89	0,87–0,93	1,08	1,02–1,14	0,94	0,90–0,98

*adjusted Odds Ratio.

**Confidence Interval (95%).

**Figure 4:**
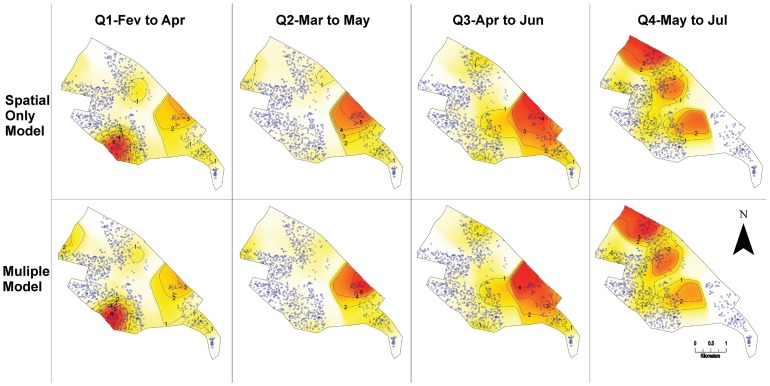
Spatial risk maps in crude and adjusted model (GAM). Legend of Figure 4: The risk maps for the occurrence of dengue cases with the crude and adjusted models for the four quarterly groupings are shown with the odds ratio values defining the color gradient ranging of white (minor value) to red (highest values). The isolines show the values corresponding to the estimates generated by the GAM model. The dots and crosses plotted on maps are showing respectively controls and dengue cases for the period.

The crude relative spatial risk model for dengue (space only) and adjusted model (space + entomological indicators) for the four quarters, obtained by GAM model are presented in Figure 4. Maps show a major variation in the transmission risk at different locations throughout the quarters, high in the south during Q1, in northeast during Q2, expanding further east during Q3 and to the north and south-central regions during Q4. Both crude and adjusted models present similar spatial feature, indicating lack of effects of the entomological indicators in the risk of disease incidence.

In Figure 3 we observed dengue cases in different areas during the study period, like “moving” through the region. Also the higher or lower intensity of the vector are identified in several areas of the study region, however it did not follow the disease displacement, suggesting that there is not a direct spatial relationship.

## Discussion

In this study, we examined the spatial and temporal distribution of three entomological indicators *A. aegypti* mosquitos – eggs, larvae-pupae, and adults – in Sumaré. Simultaneously, we analyzed the spatial distribution of dengue incidence and its association with the indicators.

The indicators presented similar seasonal behavior throughout the period and vectors were present in the entire area of the study during the dengue transmission period. Nevertheless we observed that the clusters of dengue incidence did not correspond with the distribution of entomological indicators

Incidence maps of the disease obtained from crude and adjusted models did not show significant differences in dengue incidence among the areas, suggesting that the vector indicators did not influence dengue incidence in a given space in the municipality. Although we observed a few coincidences between indicators and dengue cases in some areas, in general, the case clusters did not correspond to the highest values of vectors indices.

However, caution is required when analyzing these results, especially considering the environmental interventions carried out by the health services team during epidemic periods, such as the chemical treatment of the locations with confirmed cases, the intensification of measures to control breeding areas, and health education. In addition it should be noted that the response of the population to campaign pleas and dengue control measures, especially during an epidemic, may led to the elimination of breeding grounds of immature forms.

It is important to consider that the study area experienced infestation for over 15 years and deficient public services, besides the intense traffic between neighborhoods. The circulation of serotype DENV-1, DENV-2 and DENV-3 in the previous years may have interfered in the transmission of the virus, once a population portion may have acquired immunity to one or more serotypes. These factors may be relevant in explaining the circulation of the virus during the entire period of the study. The longstanding *A. aegypti* infestation rates in the city may have had a limited role in the circulation of the virus in Sumaré in 2011.

Thus it is plausible that the spatial distribution of the disease depends on the vector distribution, but not necessarily on its highest concentration.

Despite the evident seasonality of vector infestation and viral transmission (temporal association), the spatial distribution of disease risk and high rates of entomological indicators did not coincide in multiple models. The spatial analysis could detect risk (incidence) on a scale of blocks and neighborhoods. In this way we obtained local estimators of risk instead of mean values of indicators of large areas.

The apparent temporal correlation observed in the indicators curve over the period of transmission, was not spatially evidenced, as seen in the results of GAM models. They showed in some periods, negative association, in others, lack of statistical significance, and others risk of dengue.

Furthermore, the vector adaptability to environment inhabited by human [47] makes it difficult to control the dengue transmission, because the vectors are abundant enough for triggering and maintaining the circulation of the virus. Other variables like population size, epidemic duration and climate variables seem to determine the spread of the epidemic in longstanding infestation areas, as observed by Siqueira Junior et al. [48] and Chowell et al. [49].

The spatial association among entomological indicators has been the object of a few studies conducted at various locations. In Rio de Janeiro, Honório et al. (2009) recently reported dengue infection among residents living in areas with a low mosquito density, suggesting that the infection did not occur inside the residence [31]. In Tupã, São Paulo, Barbosa and Lourenço (2010) did not find a spatial relationship between dengue incidence and larvae infestation [28]. In Bangladesh, Ali et al. (2003) found a positive association between the dengue incidence and vector infestation in areas close to hospitals [50]. In Campinas, São Paulo, Cordeiro et al. (2011) showed a positive association between the increase in the larvae density and the incidence of dengue [34]. Chowell et al. (2008) also recorded a major variation in the weekly dengue incidence among the provinces of Peru, probably because of the level of infection spread by the mosquitoes, climate variation, circulation of different serotypes, and the population's immunological history [49].

Studies on the association between dengue transmission and entomological, environmental, socio-economical, and other indicators have presented conflicting results but reach the consensus that the dynamics of dengue transmission are complex and difficult to understand. Also authors agree that transmission depends on the environmental context and on variables that were not taken into consideration in this study, e.g., populational immunity, circulating serotypes, and control measures [14]–[16], [27], [47], [51]. Furthermore, underreporting of cases is also assumed because some patients were either asymptomatic or had mild symptoms, which were not reported to the health services, as mentioned in various studies [20], [31], [52], [53].

According to Focks et al., in order to prevent the transmission of the dengue, it is necessary to maintain vector infestation at critically low levels [54]. The Pan American Health Organization describes that a building index up to 0.1% implies low risk of dengue transmission, between 0.1 and 5%, medium risk, and above 5%, high risk [15]. However, some authors have reported dengue transmission even when the indices of entomological indicators were relatively low [14], [18]–[20]. Currently, there is no threshold for the suspicion of the risk of dengue. This study showed that the household infestation was above 5% until May, period that occurred more than 90% of the cases.

Honório et al. suggested that information on the patterns of populational movement help improve the understanding about the transmission dynamics of the disease and possible locations of its incidence [31]. Getis states that only one or few infected *A. aegypti* mosquitoes transmit the virus to several susceptible humans within a period of a few days [55]. According to Kan, the traffic of people and mosquitoes from neighborhoods where dengue incidences have been reported can explain the shift in the pattern of the epidemics [30].

This study was the first to evaluate the spatial relationship between entomological indicators of all stages of the *A. aegypti* mosquito: egg, larvae and pupae, and adult, and monthly and concurrent measurements as well as dengue incidence.

One of the limitations of the study is the lack of data about populational immunity and the interventions implemented in response to the autochthonous transmission that could have influenced the results. Besides, the population's movement patterns and the elements indicating the main transmission locations are unknown [31]. By only analyzing the reported cases during the period without considering the asymptomatic patients and those who did not seek health services, the findings were certainly underestimated. The other limitation was the use of data from a surface smoothed by ordinary kriging, which despite being a linear unbiased estimator, promotes the smoothing of results, thereby overestimating the lowest and underestimating the highest values [44].

Many factors are involved in the spatial spread of an epidemic. Certain factors, e.g., vector control programs and populational immunity to the circulating virus, may have a modulating effect on the dengue incidence. Besides, the introduction of a virus, its establishment and propagation, and the concomitant movement of various serotypes, owing to the population traffic, may also be part of the spatial dimension of the epidemic spread. In this case, infestation was not a limiting variable for transmission [32].

In this study, we were able to simultaneously analyze the incidence of dengue and conduct a survey on the entomological indicators of *A. aegypti*; however, we did not find a spatial correlation between the indicators and disease incidence. The infestation did not present a major variation in intensity and was not a limiting or determining factor of dengue incidences in a given space in the municipality. None of the different stages entomological indicators in the vector's lifecycle was a predictor of disease occurrence in areas at risk of dengue transmission. The inclusion of other variables in generalized additive models could eventually reveal other modulating factors that have an influence on spatial pattern of the disease.

## Supporting Information

Checklist S1 **STROBE checklist.**
(DOC)
